# Complete genome sequence of a Bangladesh-developed live-attenuated lumpy skin disease (LSD) vaccine strain

**DOI:** 10.1128/mra.01022-24

**Published:** 2025-01-15

**Authors:** Mohammed A. Samad, Anowar Hossen, Md. Rezaul Karim, ASM Ashab Uddin, Dipu Roy, Khairun Nahar Shithi, Mst Nazia Akter, Taposh Kumar Das, Paul Selleck, Dieter M. Bulach, Timothy R. Bowden

**Affiliations:** 1 Transboundary Animal Disease Research Center, Bangladesh Livestock Research Institute, Savar, Dhaka, Bangladesh; 2 Animal Health Research Division, Bangladesh Livestock Research Institute, Savar, Dhaka, Bangladesh; 3 Invent Technologies Ltd, Dhaka, Bangladesh; 4 CSIRO Australian Centre for Disease Preparedness, Geelong, Victoria, Australia; 5 The Asia-Pacific Centre for Animal Health, Melbourne Veterinary School, The University of Melbourne, Parkville, Australia; Portland State University, Portland, Oregon, USA

**Keywords:** complete genome sequence, lumpy skin disease virus, Bangladesh LSD vaccine

## Abstract

Lumpy skin disease (LSD) in cattle is managed through live-attenuated vaccines. Bangladesh LSD vaccine was developed from LSD virus isolate Bangladesh LSD-29 by passaging 60 times in cell culture. Here, we report the complete genome sequence of Bangladesh LSD vaccine strain.

## ANNOUNCEMENT

Lumpy skin disease (LSD) is an emerging disease in cattle throughout the world. The disease is caused by lumpy skin disease virus (LSDV; *Capripoxvirus lumpyskinpox*) (1). LSD was initially detected in Bangladesh in July 2019 (2). To assist with the disease control, the Bangladesh Livestock Research Institute has developed a live-attenuated LSD vaccine from a locally circulating strain of LSDV, which was isolated in 2021 from cattle. The LSD vaccine strain, derived from Bangladesh LSD-29 isolate (accession no. PP746705.1) (3), was sequentially passaged five times in LTC, 35 times in Vero cells (ATCC CCL 81, USA), and 20 times in MDBK cells (ATCC CCL 22, USA). After the complete monolayer of both Vero and MDBK cells, inoculation was carried out using 0.01 multiplication of infection and incubated for 1 h at 37°C. Then, the inoculum was removed and cells were maintained in MEM with 1% FBS and 5% CO2 at 37°C for 4–5 days. The viral suspension was harvested at 80% CPE by three times freezing and thawing gradually from 4°C, −20°C, and −80°C. Here, we report the complete genome sequence of Bangladesh LSD vaccine strain (accession no. PP756497.1). The complete genome sequence was subsequently determined by Next-Generation Sequencing using Illumina technology. The PureLink Viral Mini Kit (Thermo Fisher Scientific, USA) was used to extract DNA from the culture supernatant of Bangladesh LSD vaccine strain following the manufacturer's instructions. A sequencing library was prepared with Nextera DNA Flex (Illumina). Libraries had an average fragment size of 400 bp. The Illumina NextSeq 2000 platform (2 × 150 bp chemistry) was used to sequence the libraries, generating 12.80 million read pairs. The quality of raw data was assessed using FastQC v0.11.3. Trimmomatic v0.39.2 (4) was used to remove adaptors and low-quality bases from reads using the following constraints: Q score > 30, length > 35 bp, and 5′ clip = 15 (R1 and R2). Subsequently, LSDV reads were identified using Kraken2 v2.1.3 (5) and the viral collection database (version: k2 viral 20210517) and were extracted; this subset of 1.7 million LSDV read pairs (13.3% of the original read set) was assembled using SPAdes v3.13.2 with k values of 77 and 99; the final viral genome sequence was manually checked and confirmed (150,878 bp including a 2,470 bp inverted terminal repeat sequence and 161 open reading frames); and the average GC content of the genome was 25.9% (6). Using the annotation for LSDV isolate Kenya (accession no. MN072619) as a guide for product names, Prokka v1.14.6 was used to produce a draft annotation of the genome sequence (7), which was manually curated prior to submission to NCBI. Differences between the isolates, including differences in encoded proteins, were identified using Snippy v4.4.5. All tools were run with default parameters unless otherwise specified. The genomic sequences of the Bangladesh LSD vaccine and the parent strain Bangladesh LSD-29, which share 99.99% nucleotide identity, differ by one single nucleotide polymorphism at 77,637 (LSD-29; PP746705), resulting in a Ser-to-Tyr change at amino acid 111 in LSD VAC BLRI 2023 Bangladesh 086 (GenBank accession no. PP756497) (Table 1).

**TABLE 1:** Difference between Bangladesh LSD-29 isolate and Bangladesh LSD vaccine

Accession^*a*^	POS^*b*^	Type^*c*^	REF^*d*^	ALT^*d*^	NT_POS^*d*^	AA_POS^*d*^	Effect^*e*^	LOCUS_TAG	AA	Product
PP746705a	77637	SNP	C	A	332/1908	111/635	missense_variant; 332C > A, Ser111Tyr	LSDV_BLRI_029_2021_Bangladesh_086	635	70 kDa small subunit of early transcription factor

^*a*^ GenBank accession number of reference sequence.

^*b*^ POS: position of mutation with reference genome.

^*c*^ TYPE: mutation type; SNP = single nucleotide polymorphism.

^*d*^ REF: sequence in the Bangladesh LSD-29 isolate; ALT: sequence in the Bangladesh LSD vaccine isolate; NT_POS: nucleotide position; AA_POS: amino acid position.

^*e*^ Change in encoded protein; standard three letter amino acid code is used.

## Data Availability

The complete genome sequence of lumpy skin disease virus isolate Bangladesh LSD vaccine has been submitted to GenBank under accession number PP756497.1. The raw sequence reads have been submitted to the SRA database under accession number SRR29477296.
